# Unicentric castleman's disease located in the lower extremity: a case report

**DOI:** 10.1186/1471-2407-11-352

**Published:** 2011-08-12

**Authors:** Inga-Marie Schaefer, Harald Günnel, Stefan Schweyer, Michael Korenkov

**Affiliations:** 1 Department of Pathology, University Medical Center Göttingen, Germany; 2 Department of General and Abdominal Surgery, Werra-Meißner Hospital, Eschwege, Germany

**Keywords:** Castleman's disease, unicentric, mixed cellularity type, follicular dendritic cells

## Abstract

**Background:**

Castleman's disease is a rare form of localized lymph node hyperplasia of uncertain etiology. Although the mediastinum is the most common site of involvement, rare cases occurring in lymph node bearing tissue of other localization have been reported, including only a few intramuscular cases. Unicentric and multicentric Castleman's disease are being distinguished, the latter harboring an unfavorable prognosis.

**Case Presentation:**

Here, we present a case of unicentric Castleman's disease in a 37-year-old woman without associated neoplastic, autoimmune or infectious diseases. The lesion was located in the femoral region of the right lower extremity and surgically resected after radiographic workup and excisional biopsy examinations. The tumor comprised lymphoid tissue with numerous germinal centers with central fibrosis, onion-skinning and rich interfollicular vascularization. CD23-positive follicular dendritic cells were detected in the germinal centers and numerous CD138-positive plasma cells in interfollicular areas. The diagnosis of mixed cellularity type Castleman's disease was established and the patient recovered well.

**Conclusions:**

In conclusion, the differential diagnosis of Castleman's disease should be considered when evaluating a sharply demarcated, hypervascularized lymphatic tumor located in the extremities. However, the developmental etiology of Castleman's disease remains to be further examined.

## Background

Castleman's disease is regarded as a polyclonal lymphoid proliferation of unknown etiology, also designated as angiofollicular lymph node hyperplasia [1]. It was first described by Benjamin Castleman in 1954 as "localized mediastinal lymph node hyperplasia resembling thymoma" [2]. The etiology of this uncommon entity is still unclear and only few studies on the molecular and cytogenetic characteristics of this disorder exist [3,4]. The most common site of involvement is lymph node-bearing tissue with a predilection for the mediastinum (70%), but also occurring in the neck, axilla, pelvis and retroperitoeum [5]. However, a few extranodal cases have been reported in the literature, comprising 9 intramuscular cases [6]. Unicentric Castleman's disease behaves rather benign and can be cured by surgical removal, whereas the multicentric variant harbors the risk of an unfavorable course, and therefore requires an aggressive multimodal chemotherapy [1,5,6]. Here, we describe a case of unicentric mixed cellularity type Castleman's disease of the lower extremity. We present the clinico-pathological characteristics with a review of the literature.

## Case Presentation

**Figure 1:**
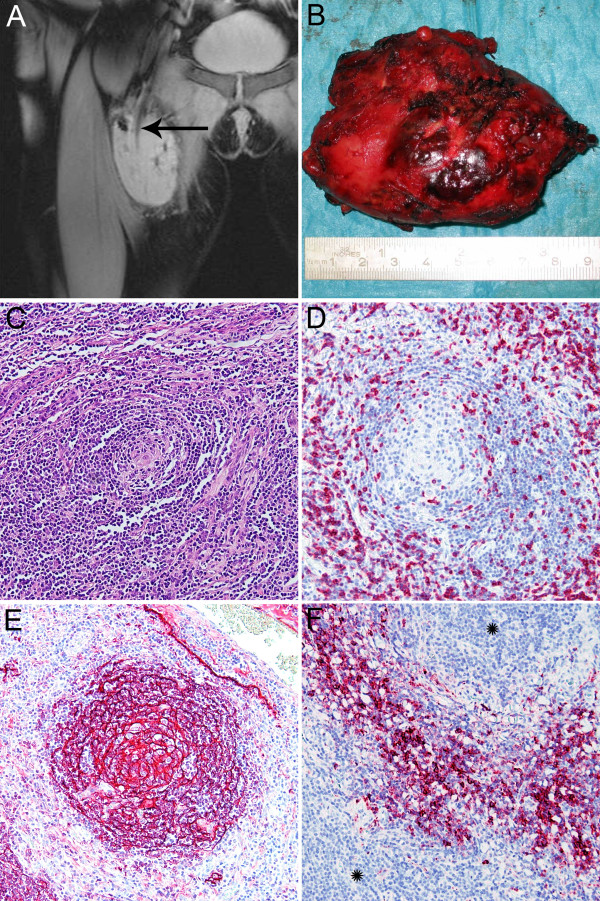
**Radiographic, gross and histopathological findings of resected unicentric Castleman's disease**. T1-weighed magnet resonance imaging revealed a sharply demarcated hyperintense mass lesion of 8.5 × 6 × 3.5 cm, located between the adductor and quadriceps femoris muscle surrounding the femoral vessels (A, arrow). Grossly, the resected tumor specimen displayed a single well-circumscribed round nodule covered by hemorrhagic soft tissue (B). On microscopic view, the tumor consisted of lymphoid tissue with numerous germinal centers with central fibrosis and marked vascularization. Some lymph follicles showed germinal centers with concentric onion skin-like layering of surrounding lymphoid cells (C). Immunohistochemical staining with CD5 revealed CD5-positive lymphocyte at the periphery of the follicles (D), whereas the follicular dendritic cells in the germinal centers expressed KiM4P (E). The spaces between the lymph follicles (stars) were filled out with CD138-positive plasma cells (F) (× 100).

A 37-year-old woman presented with a two-month history of a soft tissue mass of the femoral region of the right lower extremity. Her past medical history was uneventful and her family history exhibited no malignancies. Physical examination revealed a well-developed and nourished woman in no apparent discomfort. Examination of her heart, lungs and abdomen detected no pathologies. Palpation revealed a sharply demarcated tumor of hard consistency, fixed to the deep muscles of the medial femoral region. Laboratory findings were within normal range, except for a mild leucocytosis of 12,200 cells/μL (reference range, 4,300-11,300 cells/μL). Magnet resonance imaging (MRI) detected a homogeneous hyperintense contrast enhancing mass lesion of 9.3 cm length, located below the right inguinal ligament between the adductor and quadriceps femoris muscle compartment. The tumor showed entrapment of the femoral artery and vein, but without compression, and displayed homogeneous contrast enhancement after intravenous contrast application. These findings were suggestive of liposarcoma. Excisional biopsy of the soft tissue mass was performed and histopathological examination revealed a lymphoid tumor of unknown dignity, suggestive of either pseudo-lymphoma or B-cell lymphoma. However, a primary soft tissue tumor was ruled out. The mass lesion was closely observed for three months and a slight decrease in size to 8.5 cm was noticed by repeated MRI studies (Figure 1A). Then, the tumor was surgically completely resected and presented as a sharply demarcated mass lesion (Figure 1B) embedded in hemorrhagic soft tissue with a homogeneous brown-yellow cut surface and areas of calcification. On microscopic view, the tumor consisted of lymphoid tissue with numerous germinal centers with central fibrosis and rich interfollicular vascularization. Some lymph follicles showed the typical onion-skinning of lymphocytes concentrically surrounding germinal centers (Figure 1C), sometimes penetrated by a hyalinized blood vessel resembling a "lollipop". Other follicles displayed hyperplastic germinal centers with an infiltration of the mantle zone by mature plasma cells. Immunohistochemical staining with CD5 (DCS, Hamburg, Germany) revealed CD5-positive lymphoid cells at the periphery of the abnormal follicles (Figure 1D). Follicular dendritic cells located in the germinal centers showed positive staining for KiM4P (clone KiM4P, Kiel, Germany) (Figure 1E) and CD23 (Neo Markers, Fremont CA, USA), and numerous CD138-positive (Dako, Glostrup, Denmark) plasma cells were scattered through the interfollicular areas (Figure 1F). To test clonal immunoglobulin heavy chain gene rearrangements the indenticlone immunoglobulin heavy chain (IGH) gene clonality assay (InVivoScribe Technologies, San Diego, CA, USA) was used showing no monoclonal B-cell proliferation. Molecular testing (i. e. EBER in-situ hybridization and EBV-PCR) for Epstein-Barr virus (EBV) was negative. Histological examination confirmed complete tumor resection. Finally, the diagnosis of Castleman's disease of mixed hyaline vascular and plasma cell type (i. e. mixed cellularity type) was established and confirmed by a reference pathologist. The patient recovered well and was dismissed 5 days after the operation. No associated diseases, especially no evidence of infection with human immunodeficiency virus (HIV), could be detected. Computed tomography (CT) workup examinations after 3 months revealed no evidence of relapse or manifestations at other sites.

Written informed consent was obtained from the patient for publication of this case report and any accompanying images.

Castleman's disease is a rare disorder arising predominantly in young adult patients without a gender predominance [6]. Based on clinical and radiological findings Castleman's disease is classified as unicentric vs. multicentric disorder [5]. Only 9 cases of intramuscular Castleman's disease have been reported with a female predominance (7 women vs. 2 men), occurring between the age of 14 and 48 years [6]. Most of these cases developed in the shoulder girdle with only one case arising in the lower extremity (peroneal muscle) [6]. Here, we present a case of Castleman's disease of the lower extremity with inter-compartmental localization but probable origin in lymph nodes along the femoral artery since the tumor encased the femoral artery and vein. Whether previously described intramuscular cases developed actually from skeletal muscle or from ectopic lymphatic tissue remains to be discussed. In the present case a primary soft tissue tumor was suspected clinically. The establishment of the correct preoperative diagnosis of Castleman's disease by histopathological examination of excisional biopsies is challenging due to the rarity of this entity, especially when the tumor arises in the extremities [6]. Deep biopsy and careful tissue sampling are necessary to render the hyaline vascular parts with lymph follicles with aberrant and shrunken germinal centers with concentric onion skin-like arrangement of lymphocytes and "lollipop" formations suggestive of Castleman's disease [6]. Additionally, the characteristic hypervascularization causes homogeneous contrast enhancement in MRI and CT imaging [6]. Radiological differential diagnoses include vascular tumors, extrapleural solitary fibrous tumors, malignant lymphoma, soft tissue sarcoma and metastases [6].

Still, the etiology of Castleman's disease is poorly understood. Since monoclonal lymphocyte proliferation is not present, the disease may be of reactive nature. Clinical and radiologic findings discriminate a unicentric and a multicentric subtype. Based on histopathology Castleman's disease can be subdivided into a hyaline vascular (90%) and plasma cell type (10%) [7]. The hyaline vascular type mostly presents as a single unicentric mass harboring a favorable prognosis, whereas the plasma cell type is almost always multicentric and associated with a worse prognosis [6,7]. Altogether, the unicentric hyaline vascular type is most frequent, accounting for 72% of all cases, followed by the unicentric plasma cell (18%), and multicentric type (10%), respectively [6]. Table 1 summarizes the clinico-pathological characteristics of the different subtypes of Castleman's disease and the present case. Histopathological differential diagnoses include benign and malignant lymphoproliferative disorders such as pseudo-lymphoma, low-grade MALT (mucosa-associated lymphoid tissue)-type lymphoma, and follicular lymphoma [6]. Constitutional symptoms such as fever, night sweats and elevated interleukin-6 levels have been reported in the literature [6]. On microscopic examination the hyaline vascular type is characterized by abnormal follicles with shrunken germinal centers consisting of follicular dendritic cells, onion skinning, ingrowing hyalinized vessels, resembling a lollipop, and interfollicular hypervascularization. In contrast, the plasma cell variant typically shows hyperplastic germical centers, intact mantle zone infiltrated by mature plasma cells, and interfollicular plasmacytosis. In the present case, both features were observed, and the tumor classified as the "mixed cellularity" variant.

**Table 1:** Clinico-pathological characteristics of different subtypes of Castleman's disease as described in previous studies^1-10 ^and the present case

Histologic subtype	Predominant occurence	Epidemiology	Morphology	Immuno-histochemistry	Associated diseases	Clinical course	Therapy
Hyaline vascular	Unicentric	F = M, young adults	Abnormal follicles with shrunken germinal centers consisting of FDC, "onion skinning", vascular ingrowth: "lollipop" formations, interfollicular hypervasculariza-tion	FDC: CD21, CD35, EGFR	Nephrotic syndrome, mixed connective tissue disorder, Hodgkin disease	Benign	Complete resection
Plasma cell	Multicentric	F = M, young adults	Hyperplastic germical centers, intact mantle zone infiltrated by mature plasma cells, interfollicular plasmacytosis	Plasma cells: CD138	Elevated IgG4, elevated IL-6; infections: HHV-8, HIV; autoimmune, paraneoplastic and connective tissue diseases	Aggressive	Multimodal approach: radiation, chemo-therapy, and/or surgery
Mixed cellularity (present case)	Unicentric	F, 37 years	A combination of hyaline vascular and plasma cell type	FDC: CD23, KiM4P; Plasma cells: CD138	None	Benign	Complete resection

FDC: Follicular dendritic cells.

The developmental etiology of Castleman's disease is still being discussed. In the hyaline vascular type CD5-positive lymphocyte proliferations, possibly stimulated by certain cytokines, have been suggested to play a role in the development of the disease [8]. Some authors even designate this subtype as an "atypical CD5-positive B-cell disorder" whereas others propose a disease of follicular dendritic cells [8,9]. In our case, CD5-positive B-cells were detected in the periphery of the germinal centers surrounding the follicular dendritic cells. Cytogenetic anomalies detected in stromal cells in hyaline vascular Castleman's disease include t(1;22)(qter;q13) and t(7;8)(q37.3;q12) [3]. Furthermore, rearrangement and partial deletion of the *HMGIC *gene in follicular dendritic cells due to an unbalanced 6;12 translocation has been reported, suggesting a clonal proliferation of follicular dendritic cells the hyaline vascular subtype [9]. Some reports describe a possible association with the development of angiomyoid proliferative lesions and vascular tumors and a possible mesenchymal tumorigenesis being implicated in hyaline vascular Castleman's disease [9]. An association of the multicentric type with an human herpes virus (HHV)-8 infection in HIV-positive patients has recently been discovered [4]. HIV, other conditions of immune deregulation and primary auto-immune diseases (systemic lupus erythematosus, POEMS syndrome, nephrotic syndrome) may even exhibit histologic findings similar to Castleman's disease, including regressive lymph nodes [5,8]. Furthermore, excessive production of interleukin-6 in germinal centers of multicentric Castleman's disease has been observed to be associated with an HHV-8 infection and it has been suspected to play an important role in the pathophysiology [5,7,10]. However, in the present case no constitutional symptoms or related disorders could be observed as yet.

## Conclusions

Although infrequent, it is important to bear the differential diagnosis of Castleman's disease in mind when evaluating a sharply demarcated, hypervascularized lymphatic hyperplasia located in the extremities. The etiology of Castleman's disease remains to be further examined in future studies.

## Competing interests

The authors declare that they have no competing interests.

## Authors' contributions

IMS and SS performed the histopathological, immunohistochemical and genetic examinations and established the diagnosis. IMS, SS and MK participated in the design of the study. IMS, HG, SS and MK participated in writing the manuscript. HG and MK treated and observed the patient, including follow-up. IMS, HG, SS and MK acquired the radiographic, gross and histological pictures, and all authors read and approved of the final manuscript.

## Pre-publication history

The pre-publication history for this paper can be accessed here:

http://www.biomedcentral.com/1471-2407/11/352/prepub
